# Case report – ancient schwannoma of the scrotum

**DOI:** 10.1186/1471-2490-7-1

**Published:** 2007-01-23

**Authors:** Peter T Chan, Sankalp Tripathi, Su E Low, Lee Q Robinson

**Affiliations:** 1 Surgical Directorate, Warrington Hospital, Lovely Lane, Warrington, UK; 2 Department of Radiology, Warrington Hospital, Lovely Lane, Warrington, UK; 3 Department of Pathology, Warrington Hospital, Lovely Lane, Warrington, UK

## Abstract

**Background:**

Scrotal schwannoma is a rare neoplasm and poses a diagnostic challenge to urologists. This article describes a rare case of ancient scrotal schwannoma and reviews the current modality of investigation and treatment of this tumour.

**Case report:**

A 28 year old man presented with a 3-month history of an asymptomatic scrotal swelling. Ultrasonography and computer topography revealed an intra-scrotal and extra-testicular mass without local invasion. Surgical excision was undertaken and histology was an ancient schwannoma of the scrotum.

**Conclusion:**

Schwannoma is a benign encapsulating neoplasm with an overall low incidence, occurring mostly in the head and neck region and seldom in the scrotum. Histology shows two distinctive patterns, Antoni type A and B areas. Variations of schwannoma such as cellular, ancient, glandular and epithelioid are observed based on the appearances. Ancient schwannoma exhibits pleomorphism without mitosis as the result of cellular degeneration, which can lead to an erroneous diagnosis of malignancy. Imaging modalities are non-specific for schwannomas, but can define tumour size, site and extension. The mainstay treatment is complete excision, although local recurrence may occur in large and incompletely excised lesions. Malignant change is exceedingly rare.

## Background

The "*ancient*" variant of schwannomas is a rare subtype of a benign encapsulated neoplasm of the nerve sheath [1]. A review of current literature has revealed several reported sites but not in the scrotum. Here we report such a scrotal tumour arising in a 28-year old man, paying particular attention to the potential diagnostic difficulties prior to surgical intervention.

## Case report

A 28-year old man presented with a three month history of an asymptomatic scrotal swelling. He had previously undergone orchidopexy in childhood for a mal-descended right testis. There is otherwise no other significant medical or family history. The mid-scrotally located mass measured 4 × 7 cm and was nodular, hard and clinically separate from the testes. It was not attached to the scrotal skin or other underlying structures. Lymphadenopathy was not detected.

**Figure 1:**
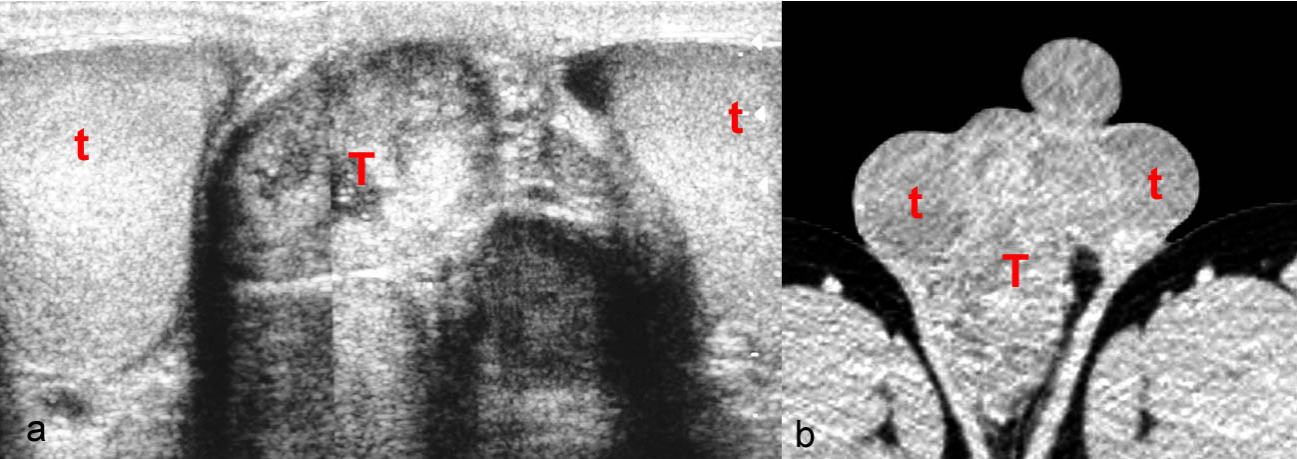
**Imaging of scrotal ancient schwannoma**. a) Ultrasound image and b) CT image showing an intra-scrotal and extra-testicular mass in the mid-scrotal region [Capital red T – tumour, red t – testes].

Initial ultrasonography (Fig 1a) showed a large extra-testicular and multinodular mass in the midline of the scrotum. The largest nodule, located behind the right testis was cystic. Both testes appeared normal. Subsequent staging Computer Tomography (CT) of the scrotum, abdomen and chest was performed, which identified a similarly localised heterogeneous mass of 6.9 × 3.7 cm, separate from the testes (Fig 1b) and did not extend into the tunica vaginalis. There was no evidence of distant or nodal metastases.

An initial diagnostic wedge biopsy indicated the lesion could be an ancient schwannoma. Curative surgical excision with partial scrotectomy was undertaken for removal of the mass and for definitive diagnosis. At resection, the tumour appeared to be superficial to the tunica vaginalis, testes and corpus spongiosum. Inferiorly, the tumour was attached to the bulbospongiosus requiring partial resection of the superficial muscle fibres.

**Figure 2:**
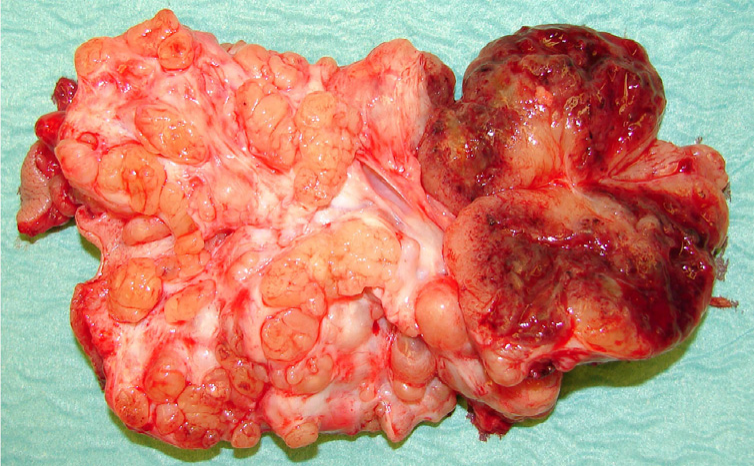
**Macroscopic appearance this scrotal ancient schwannoma**. A white multinodular tumour with haemorrhagic areas.

**Figure 3:**
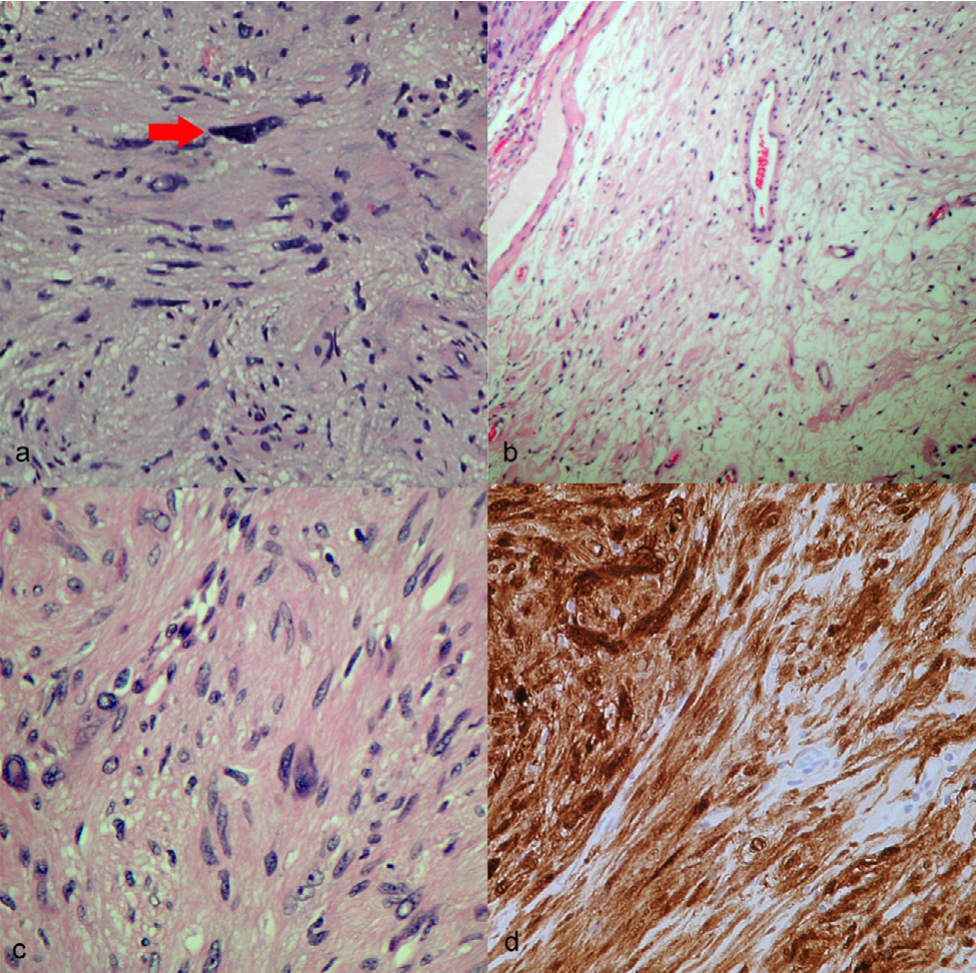
**Microscopic appearance of ancient schwannoma**. a) spindle cells in an Antoni type A area; a red arrow indicates a large bizarre hyperchromatic nucleus b) Antoni B areas consisting of spindle cells within a loose myxoid matrix, c) occasional pleomorphic nuclei within Antoni A areas, and d) positive S100 immunohistochemical staining.

The resected specimen (Fig 2) was white and multinodular, with areas of cystic change and haemorrhage. Microscopically (Fig 3), proliferation of spindled shaped cells with fibrillary cytoplasm was seen, with dense fibrous bands arranging the cells into nodules. In some cells, marked nuclear hyperchromaticism and atypia were seen. Mitoses were not present. Within the lesion, cellular areas were interspersed with looser myxoid and cystic areas. Blood vessels with thickened hyalinised walls were noted. Staining for S100 protein was positive in the tumour cells. The histological features are that of an ancient schwannoma. The margins of this specimen were ragged and the completeness of excision was difficult to assess.

## Conclusion

A schwannoma is a benign encapsulated neoplasm derived from schwann cells of the nerve sheath. The exact incidence of schwannomas is unknown, but they are rare. Schwannomas are found in all age groups but are more common in the first four decades and affect both sexes equally. They may occur in association with neurofibromatosis or arise sporadically. The microscopic appearance of schwannoma is distinctive [1], with two recognisable patterns. Antoni A areas are composed of compacted spindle cells often arranged in palisades or in an organoid arrangement (Verocay bodies). Antoni B areas consist of tumour cells suspended in a myxomatous matrix that may appear microcystic. Several variants of schwannomas based on appearances have been observed, including ***cellular, glandular***, ***epithelioid ***and ***ancient ***types, and all exhibit benign progression. Cellular schwannomas are almost exclusively composed of Antoni A areas but lack Verocay bodies. The glandular and epitheloid variants compose of epitheloid areas and glandular component, respectively, to acquire their descriptive names. ***Ancient schwannomas ***show bizarre hyperchromatic nuclei without mitoses. To the unwary, these features can lead to an erroneous diagnosis of malignancy, although the very low mitotic activity should allow these tumours to be distinguished from malignant nerve sheath tumours.

A literature review showed that whilst ancient schwannomas are rare, most cases occur in the head and neck region [2] (trigeminal nerve, facial nerve, vestibular nerve, vagus nerve, parotid, thyroid, vocal cord, floor of mouth, orbit and infra-temporal fossa). Other less common sites include the extremities, mediastinum, thorax [3], retroperitoneum [4], pancreas [5] and pelvis [6]. Scrotal schwannomas have been infrequently described in the medical literature [7,8,10], however, we could not find references pertaining to the ancient variant in the scrotal region.

Schwannomas pose a difficult diagnostic challenge to urologists. Radiological findings are often non-specific [9]. Ultrasonography can differentiate between solid and cystic tumours. CT can be helpful in determining the size, location, local involvement and distant spread. Magnetic resonance imaging (MRI) provides similarly useful information as CT, but yields better visualisation of the tumour. Fine needle aspirate (FNA) cytology is not often helpful because the tissue architectural information required is not obtainable from cytological specimen [4]. The only gold standard diagnostic investigation is histology of either biopsy or excised specimen.

Surgical excision has remained the mainstay of treatment. Although benign, large and incompletely excised lesions are capable of recurrence; malignant change is exceedingly rare [3,10]. This patient will require a prolonged period of surveillance due to the large size of the tumour and the uncertainty of complete excision.

## Competing interests

The author(s) declare that they have no competing interests.

## Authors' contributions

PTC drafted the manuscript, prepared illustrations and performed the literature search. ST helped to draft the manuscript and helped to acquire ultrasound and CT images. SEL helped to draft the manuscript, paying particular attention to the pathological aspect and kindly acquired histological images for illustration. LQR conceived this study and supervised the drafting and overall structure of the manuscript. All authors read and approved the final manuscript.

## Pre-publication history

The pre-publication history for this paper can be accessed here:
